# Characterizing nutrient patterns of food items in adolescent diet using data from a novel citizen science project and the US National Health and Nutrition Examination Survey (NHANES)

**DOI:** 10.3389/fnut.2023.1233141

**Published:** 2023-09-21

**Authors:** Jonah T. Treitler, Senait Tekle, Jennifer Ushe, Linda Zanin, Teri Capshaw, Gregory Tardieu, Alexander Libin, Qing Zeng

**Affiliations:** ^1^Thomas Jefferson High School for Science and Technology, Alexandria, VA, United States; ^2^The Biomedical Informatics Center, George Washington University, Washington, DC, United States; ^3^Alexandria City Public Schools, Alexandria, VA, United States; ^4^Georgetown-Howard Universities Center for Clinical and Translational Science (GHUCCTS), Washington, DC, United States

**Keywords:** adolescents, citizen science, nutrients, diet, cluster analysis, schools

## Abstract

**Introduction:**

A healthy diet is essential for promoting good health during adolescence and mitigating disease risks in adulthood. This underscores the need for improved nutrition education and increased access to healthier food choices. However, the accuracy of dietary data poses a significant challenge in nutritional research.

**Methods:**

We utilized and analyzed a novel dietary record dataset collected through a high school citizen science project to address this issue. We focused on nutrients rather than food groups to characterize adolescent dietary patterns. The same analyses were performed on the 2019–2021 National Health and Nutrition Examination Survey data for comparison.

**Results:**

Based on the U.S. Food and Drug Administration’s recommended daily value (DV) for nutrients, the majority of food items in our citizen science dataset are low (i.e., <5% DV) in lipids, fiber, potassium, calcium, iron, sugar, and cholesterol. Only a minority of items are high (i.e., >20% DV) in macro and micronutrients. The clustering analysis identified nine food clusters with distinct nutrient profiles that vary significantly in size. The analyses on the NHANES data yielded similar findings, but with higher proportions of foods high in energy, lipids, carbohydrates, sugar, iron, and sodium compared with those of the citizen science dataset.

**Discussion:**

This study demonstrates the potential of citizen science projects in gathering valuable dietary data and understanding adolescent nutrient intake. Identifying critical nutrient gaps can guide targeted nutrition education and the provision of accessible healthier food options, leading to positive health outcomes during adolescence and beyond.

## 1. Introduction

During adolescence, young individuals experience rapid growth and development, making this a crucial period wherein proper nutrition is essential in ensuring optimal wellbeing. Consuming well-balanced meals rich in fruits, vegetables, whole grains, and lean proteins can help maintain a healthy weight while reducing the risk of developing chronic diseases and promoting overall physical health (1). Adopting healthy behaviors, such as adhering to a balanced diet, can also contribute to better mental wellbeing, such as improved mood, reduced stress and anxiety levels, and greater self-esteem (2). A balanced diet not only provides the necessary nutrients but also supplies the energy to support physical activity, psychological health, and academic performance, leading to better concentration and increased productivity. Adolescents who follow such diets may experience better sleep quality, which also positively impacts both their physical and mental health (3).

However, multiple prior studies have indicated a need for improved nutrition education and better access to healthier food choices for adolescents. A meta-analysis of Global School-Based Student Health Surveys conducted in 2018 found that adolescents’ diets are composed of an excess of processed foods, sugars, and saturated fat, while showing low intake of fruits, vegetables, and whole grains, with wide variability by subpopulation. This highlights the need for interventions that promote healthy behaviors and reduce the prevalence of health risk behaviors among adolescents, including education on the importance of healthy eating (4). Furthermore, a systematic review examining the relationship between diet and mental health reported evidence of a significant, cross-sectional relationship between unhealthy dietary patterns and poorer mental health in children and adolescents (5). Additionally, poor dietary habits have been linked to various diseases and conditions, including headache (6), diabetes (7), and breast cancer (8). The findings from these studies highlight the need for improved nutrition education and access to healthier food choices among adolescents.

Despite these findings, it has been widely described that self-reported dietary studies may be affected by measurement error (9), potentially leading to a misrepresentation of adolescents’ actual dietary habits. To address this issue, multiple dietary assessment methods have been developed, each exhibiting distinct strengths and weaknesses (10). Among these methods, the dietary record approach is one of the standard methods, although it places a relatively significant burden on participants and requires high motivation (11). Food Frequency Questionnaires (FFQ), on the other hand, offer ease of implementation but suffer from low accuracy (12). Another viable option is the twenty-four-hour dietary recall, which generates detailed data with a lower participant burden; however, it requires trained interviewers, making it expensive and time-consuming (13).

In a novel citizen science project, students from the T.C. Williams High School [now Alexandria City High School (ACPS)] in Virginia successfully collected detailed lifestyle data pertaining to their diet, physical activity, and sleep. Collaborating in groups of two to five students, each group selected a specific research focus after receiving clear guidelines on the data collection process. Working closely with both George Washington University faculty and ACPS staff, the students ensured the data quality, accuracy, and reliability. This close working relationship was vital in generating a valuable dataset and yielding valuable insights into adolescent lifestyles. The approach, which involves citizens’ participation in scientific projects, can mutually benefit study subjects and researchers. By acting as both the study subject and researcher, data source and data analyst, the students were actively engaged in the study and played a central role in formulating the research questions of interest to them. Citizen science projects have been shown to offer several benefits to the scientific community and the advancement of scientific knowledge. Silvertown (14) describes the emergence and appeal of citizen science as a valuable approach to scientific research. The paper highlights how citizen science can benefit scientific research by augmenting data collection, engaging the public, building community involvement, providing access to data, enabling interdisciplinary collaboration, and improving scientific understanding. The author highlights that citizen science can help overcome limitations in traditional research methods and foster collaboration between scientists and the public (14).

The primary aim of this project was to actively engage students in scientific research and empower them with a deeper understanding of the scientific process. Through their involvement as researchers and citizen scientists, the students gained a sense of ownership over the project. They contributed to a unique data source that provides valuable insights into adolescent diets. Moreover, the inclusive nature of the data collection, which encompassed both school and non-school days, increased the representativeness of the data, making it more reliable and informative.

This study analyzes a dataset of 3,948 food items recorded by citizen scientists and finds that today’s U.S. adolescent diet includes a wide range of food items traditionally viewed as “ethnic food.” As such, the high schoolers’ diet included a diverse collection of items such as “Acheke,” “Fried Bami,” and “Japchae.” However, categorizing these items by broad food groups such as fruits, vegetables, grains, protein, or dairy cannot fully capture their varying nutritional profiles. Therefore, to better represent and characterize the adolescent dietary patterns, we performed a clustering analysis of the food items based on their macronutrients and micronutrients. This approach enabled us to capture a more nuanced understanding of the diet of adolescents. To the best of our knowledge, no previous clustering analysis has been performed on adolescent dietary data, as prior clustering analyses mainly focused on food items and food groups to understand adult dietary patterns (15).

To provide a comparative analysis, we identified the adolescent population (15–17 years old) in the 2019–2021 National Health and Nutrition Examination Survey (NHANES) dataset and repeated the analysis. NHANES utilizes trained dietary interviewers fluent in Spanish and English to conduct 24-h dietary recall interviews (16). To ensure data accuracy, NHANES also performs a follow-up dietary interview via telephone 3–10 days after the initial in-person recall for all participants when possible. Additionally, NHANES utilizes “a complex, multistage, probability sampling design to select participants representative of the civilian, non-institutionalized U.S. population” (17).

## 2. Materials and methods

### 2.1. Datasets

The citizen science project was reported in a prior publication (18). We briefly describe the data collection here. The data was collected from 28 high school students who participated in the study to self-report their healthy lifestyle behaviors and mood. Data collection took place from December 2018 to January 2019. The students who collected data were given detailed instructions by their teacher and the George Washington University researchers throughout a biotechnology course. Participants in the study filled out a one-time questionnaire and a daily mood tracker for 30 days and used fitness trackers to monitor their daily activity, sleep, and steps. The students conducted literature reviews, developed research questions and hypotheses, and collected data on mood, activity levels, sleep, and nutrition using surveys and fitness trackers. The dataset included demographic information, such as age, gender, and race/ethnicity, as well as data on their reported behaviors and mood, perceptions of the project, and its impact on their understanding. The study incorporated principles of citizen science, with students actively participating in the research process and contributing to the design and implementation of the study. By design, the citizen science dataset is a convenient sample.

From the NHANES data, we identified 735 adolescents (15–17 years of age) from 2017 to March 2020. NHANES refers to this dataset as the pre-pandemic data. The NHANES survey follows a multi-year, stratified, clustered four-stage design. The stages included: “(a) primary sampling units (counties, groups of tracts within counties, or combinations of adjacent counties), (b) segments within primary sampling units (census blocks or combinations of blocks), (c) dwelling units (households) within segments, and (d) individuals within households” (19). In addition, during the study period, we selected individuals with the desired age group we were interested in. It is important to note that NHANES incorporated oversampling techniques to ensure adequate representation of minority groups (Hispanic, non-Hispanic black, and non-Hispanic, non-black Asian) as well as low-income individuals (at or below 185% of the federal poverty level).

### 2.2. Citizen science data preparation

To prepare the data for analysis, we first identified the food items from the dietary record. The citizen science data required significant cleaning, e.g., there were misspellings and concatenations of different food items. We then utilized the nutritional database tables from the United States Department of Agriculture (USDA) (20) as the primary data source for nutrient information for each food item. It is worth noting that the NHANES database also used the USDA to calculate the food energy and nutrient data. We used alternative nutritional data sources such as Nutritionix, Daily Value, and other websites for food items we could not find in USDA. Due to the variation in serving sizes not only by food items but also by brand and data source, we followed the USDA’s practice of using 100 g as the standard serving size for all food items.

Finally, to ensure data accuracy, we thoroughly examined the dataset to identify clear errors, missing values, and duplicate records. Examples of errors include instances where a food item contained over 100 g of an individual component in 100 g of food, missing carbohydrate values from a regular pasta product, or identical food items with different names. To correct these errors, we utilized the previously mentioned alternative data sources.

### 2.3. NHANES data preparation

National Health and Nutrition Examination Survey collects data on each participant’s specific food items, along with their corresponding mass and nutrient values. To ensure that our analyses of the NHANES data were consistent with those of the citizen science data, we standardized the nutrient values to a 100 g serving size. This enabled us to make meaningful comparisons between the two datasets and draw accurate conclusions.

### 2.4. Data analysis

To analyze the energy and nutrient content of the food items, we calculated the minimum, mean, maximum, and standard deviation for all food items in the dataset. We also determined the percentage of food items with high and low values based on the Food and Drug Administration (FDA) definition. Please note that the data analyses was carried out on the food items, not patients. There are 3,948 food items in the citizen science dataset and 11,430 in the NHANES dataset.

To identify clusters of similar food items, we chose two widely used methods: K-Means (21) and Gaussian Mixture Modeling (GMM) (22). K-Means is a vector quantization method that assigns a data point to one of the k clusters with the nearest mean (i.e., the cluster center or centroid). The objective function is:

$$J=\sum_{i=1}^{m}\sum_{k=1}^{K}w_{i\,k}\,{∥x^{i}-\mu_{k}∥}^{2}$$

Where: K = number of clusters
*x^i^* = represents data point
*m* = number of points
*w*_*ik*_ = 1 if the data point (*x^i^*) belongs to the cluster (k)
*w*_*ik*_ = 0 if the data point (*x^i^*) does not belong to the cluster (k)
μ_*k*_ = denotes the centroid of xi’s cluster

GMM is a statistical method that assumes all data points are generated from n underlying Gaussian distributions. Both K-Means and GMM require a predefined number of clusters n. K-Means seeks to minimize the within-cluster variance, and GMM seeks to maximize the model’s fit (i.e., the probability that the model generates the observed data). The probability distribution function of Gaussian Distribution with d features is defined as:

$$N\,\left(\mu ,\Sigma \right)=\frac{1}{{\left(2\,\pi \right)}^{d/2}\,\sqrt{\left|\Sigma \right|}}\,e\,x\,p\,\left(-\frac{1}{2}\,{\left(x-\mu \right)}^{T}\,\Sigma^{-1}\,\left(x-\mu \right)\right)$$

Where: μ = Mean
Σ = Covariance Matrix of the Gaussian
d = The number of features in our dataset
x = the number of data points

There are several methods for estimating the number of clusters. One of the most widely used methods is the Bayesian Information Criterion (BIC), which balances the model complexity and fit. This BIC is calculated as follows:

$$B\,I\,C=k\,l\,n\,\left(n\right)-2\,\text{ln}\,\left(\hat{L}\right)$$

Where: $\hat{L}$ = the maximized value of the likelihood function of the model M, i.e., $\hat{L},p\,\left(x|\hat{\theta },M\right),$where $\hat{\theta }$ are the parameter values that maximize the likelihood function
*x* = the observed data
*n* = the number of data points in *x*, the number of observations, or equivalently, the sample size
*k* = the number of parameters estimated by the model.

After using the BIC, we confirmed the cluster number using the “Elbow Method,” a graph-based method. In the elbow method, the sum of the square distance between points in a cluster and the cluster centroid is plotted against the number of clusters k, forming a curve. Before the elbow point, the slope is much steeper than that after the elbow. We first determined the number of clusters using the unique food items in the citizen science project and then applied it to both the citizen science and NHANES datasets.

Since the range of values varies greatly by nutrient, we scaled the nutritional data before clustering. For the resultant clusters, we calculated the cluster centers/centroids, and the nutrient values of the centers were normalized using the FDA-recommended daily values. All data analysis in this study was performed using R (23).

## 3. Results

### 3.1. Participants characteristics

The citizen science project had a relatively small number of participants with an observation period of over 1 month. In contrast, NHANES had a larger number of patients but a shorter observation period of only 2 days per person (Table 1). It is worth noting that NHANES does not differentiate race for Hispanic participants.

**TABLE 1:** Demographics of study participants in the citizen science and NHANES datasets.

	Citizen science (*N* = 28)		NHANES (*N* = 735)	
	Mean/N	Std/%	Mean/N	Std/%
Age	16.5	0.81	16	0.81
Gender				
Female	18	64.3%	353	48.0%
Male	10	35.7%	382	52.0%
Race				
White American	21	75.0%	227	30.9%
African American	3	10.7%	181	24.6%
Asian	3	10.7%	84	11.4%
Others	1	3.6%	80	10.9%
Ethnicity				
Non-Hispanics	n/a	n/a	572	77.8%
Hispanics	n/a	n/a	163	22.2%

### 3.2. Descriptive statistics of energy and nutrients

**FIGURE 1:**
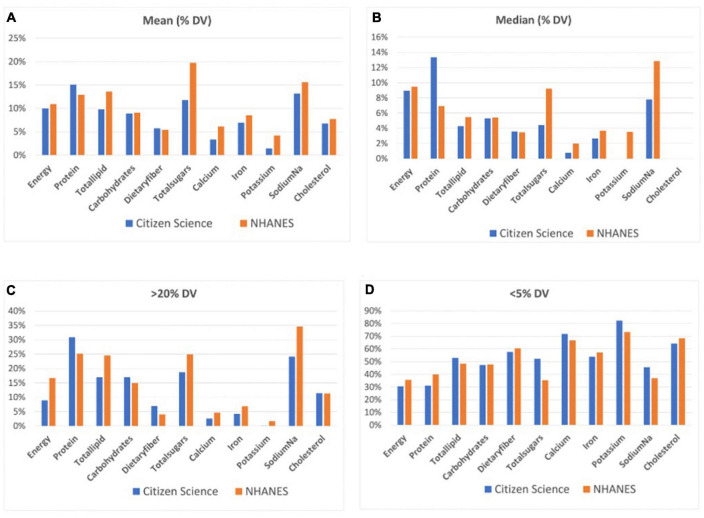
The mean **(A)** and median **(B)** energy and nutrients of food items as well as the percentage of food items exceeding the 20% **(C)** and below the 5% **(D)** thresholds in the Citizen Science and NHANES datasets.

We applied the FDA’s rule of classifying food items with less than 5% of Daily Value (DV) of any particular nutrient as low and those with greater than 20% per serving as high. The analysis showed that the citizen science data had low median and mean values of multiple nutrients: Median total lipids, dietary fiber, total sugar, calcium, iron, potassium, and cholesterol, and mean calcium and potassium were low. In the NHANES data, the median dietary fiber, calcium, iron, potassium, cholesterol, and mean potassium were low. None of the means and medians were high (Table 2; Figure 1).

**TABLE 2:** Citizen Science and NHANES energy and nutrients descriptive statistics, including the range, median, and 1*st* and 3*rd* quatiles of the nutrient values which are normalized as the percentage of the FDA DV.

	Energy	Protein	Total lipid	Carbo-hydrates	Dietary fiber	Total sugars	Calcium	Iron	Potassium	Sodium	Cholesterol
Citizen science (Number of food items = 3,948)											
Min.	0%	0%	0%	0%	0%	0%	0%	0%	0%	0%	0%
1st Qu.	4%	4%	0%	2%	0%	0%	0%	0%	0%	1%	0%
Median	9%	13%	4%	5%	4%	4%	1%	3%	0%	8%	0%
Mean	10%	15%	10%	9%	6%	12%	3%	7%	1%	13%	7%
3rd Qu.	15%	23%	13%	12%	9%	14%	5%	9%	3%	19%	10%
Max.	45%	126%	128%	37%	102%	188%	74%	185%	29%	400%	162%
>20%	9%	31%	17%	17%	7%	19%	3%	4%	0%	24%	11%
<5%	31%	31%	53%	47%	58%	52%	72%	54%	82%	46%	64%
**NHANES (number of food items = 11,430)**											
Min.	0%	0%	0%	0%	0%	0%	0%	0%	0%	0%	0%
1st Qu.	3%	2%	0%	0%	2%	0%	3%	1%	1%	2%	1%
Median	9%	7%	5%	5%	3%	9%	2%	4%	4%	13%	0%
Mean	11%	13%	14%	9%	5%	20%	6%	9%	4%	16%	8%
3rd Qu.	17%	20%	20%	14%	8%	20%	8%	9%	5%	24%	9%
Max.	45%	156%	128%	36%	123%	200%	106%	193%	76%	342%	338%
>20%	17%	25%	25%	15%	4%	25%	5%	7%	2%	35%	11%
<5%	36%	40%	48%	48%	60%	35%	67%	57%	73%	37%	68%

In the citizen science data, the percentages of food items that were categorized as high or low in energy or in nutrients differed. A high percentage (31%) of food items have high protein, while 0% have high potassium. There is a higher percentage of food items with low values on energy and almost all nutrients: 82% have low potassium while 31% have low energy and protein. A similar pattern is observed in the NHANES data. Both datasets have high percentages of food items low in fiber, calcium, potassium, iron, and cholesterol (Table 2; Figure 1).

### 3.3. Cluster analyses

**FIGURE 2:**
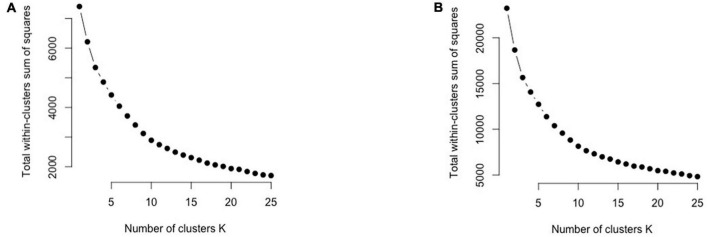
The elbow plots for the Citizen Science **(A)** and GMM **(B)** cluster analyses to help determine the number of clusters.

**FIGURE 3:**
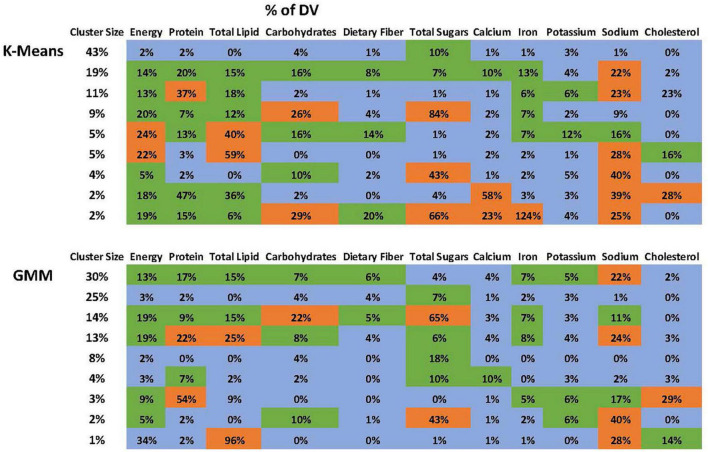
Nutrient profile of the clusters generated using the ACPS Citizen Science project dataset. The profiles from the K-Means and GMM are not identical but have many similarities.

**FIGURE 4:**
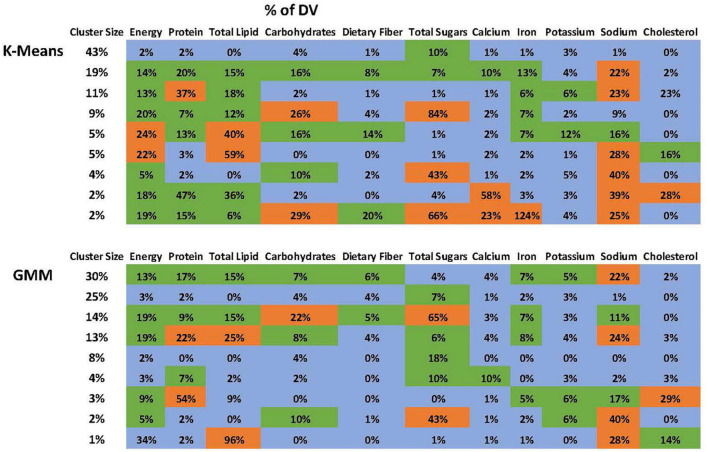
Nutrient profile of the clusters generated using the ACPS Citizen Science project dataset. The profiles from the K-Means and GMM are not identical but have many similarities.

The optimal number of clusters based on BIC is 9 (Figure 2). Figure 2 demonstrates that “9” is in the “elbow area.” The energy and nutrient profiles of the clusters differ somewhat based on the clustering method and dataset (Figures 3, 4). The largest cluster of food items generated from using both K-means and GMM from the citizen science project is low in energy and all nutrients except sugar. There are also several other large clusters, one of which is high in protein and has an amount of sodium in the upper range of normal. Another cluster is high in carbohydrates and normal levels of energy, protein, and iron.

The NHANES analyses also yielded a low-energy cluster that included all nutrients except for sugar. Other large clusters include one high in both protein and sodium, one high only in sodium, and one high in carbohydrates and sugar.

## 4. Discussion

### 4.1. Findings

This study analyzed data from a novel citizen science project and a national survey to examine the nutrient content of food items consumed by adolescents in the US. Our results show that the majority of food items in our citizen science dataset are low (i.e., <5% DV) in lipids, fiber, potassium, calcium, iron, sugar, and cholesterol, and only a minority of items are high (i.e., >20% DV) in any macro or micronutrients. The findings from NHANES differ slightly, with most food items low in fiber, potassium, calcium, iron, and cholesterol. Only a minority of items are high in any macro and micronutrients.

The clustering analyses yielded nutrient profiles that provide a new characterization of adolescent dietary patterns in the US. The analyses identified a large cluster low in energy and nutrients, except for the sugar found in both datasets. Each dataset also has a large cluster that is high in protein and high/borderline high in sodium. The citizen science dataset had a large cluster with high carbohydrates, while the NHANES dataset had a cluster with high carbohydrates and sugar.

### 4.2. Implications

Citizen science is a valuable addition as a new data source and can supplement and complement established data sources like NHANES. Our data analyses showed that the results from the citizen science project and NHANES are similar but not identical. NHANES is highly respected and widely used but has limitations. For example, the NHANES data relies solely on USDA data for nutritional information, which may not capture certain “ethnic” foods or branded products. Working directly with the citizen science data, we can obtain diverse nutritional information on ethnic foods and certain other products from alternative sources when needed.

However, it is important to note that compared to existing dietary collection methods, the citizen science approach may have higher data collection burdens on participants, although their motivation to participate may also be higher. Conversely, the cost of obtaining data from citizen science is lower as participants are not research subjects that require compensation.

It is well known that, on average, US adolescents do not consume enough fruits, vegetables, and whole grains. Some studies have examined trends in specific nutrient intake like sugar, fiber, or potassium along with the associated health outcomes (24, 25). However, few studies have attempted to characterize multi-nutrient patterns through clustering, and none have been carried out in adolescents. As such, our analysis provides new insights into the dietary patterns of US adolescents and can serve as a foundation for further research in this area.

### 4.3. Limitations

There are several limitations to this study. First, while the citizen science project provided valuable data on adolescent dietary patterns, the sample was relatively small, which may limit the generalizability of our findings. Additionally, the longer observation period per person may have increased the risk of recall bias or other sources of bias. Further research with a larger and more diverse sample size is needed to validate our findings. There is no standard formula for calculating sample size. Some literature suggested that each cluster should have at least 20–30 samples.(26) In this study, the number of unique subjects is modest but the number of food items being analyzed is much larger: *n* = 3,948 from citizen science and *n* = 11,430 from NHANES. As a result, most clusters had considerably more than 30 samples.

Second, we did not collect serving size information in the citizen science project. This is because participants found it particularly burdensome to estimate the amount of food consumed accurately. This could have affected our ability to accurately assess nutrient intake and make meaningful comparisons with NHANES data. Future studies should consider methods to improve the accuracy of serving size estimation.

Third, citizen scientists are not trained professionals. Despite their strong motivation, their recall is not perfect, and their dietary record had varying degrees of details, e.g., an entry is just “fries” while another is “Wendy’s chili cheese fries.” While we provided training and guidance, it is possible that some participants struggled to accurately recall and record their dietary intake. More rigorous training and quality control measures for future citizen science projects could improve data quality.

Fourth, clustering analysis results are affected by the chosen method and the number of clusters. While BIC is commonly used, there are alternative methods. We observe that larger clusters are often more stable across different methods and cluster numbers, while smaller ones can be significantly different. Future studies could explore different clustering methods and assess the stability of the resulting clusters.

### 4.4. Future work

We plan to correlate different nutritional patterns with health outcomes in future studies. We are also interested in exploring the correlation between nutritional patterns, demographic backgrounds, and consumer behavior. By examining these associations, we hope to gain a deeper understanding of the complex interplay between diet and health and identify potential avenues for targeted interventions to improve dietary habits and health outcomes among adolescents.

## 5. Conclusion

This study analyzed data from both a citizen science project and the NHANES sample to identify nutritional patterns in the diets of US adolescents. Our findings suggest that the majority of food items consumed by this population are low in nutrients, including fiber, potassium, calcium, iron, and cholesterol. The largest cluster of food items is low in energy and nutrients, with the exception of sugar. These results highlight the need for targeted interventions to improve the dietary habits of this population. Lifestyle change can be difficult and slow. While health is part of the standard current curriculum in secondary schools across the US, there is clearly room for improvement.

Furthermore, our study suggests that citizen science data could be a valuable addition to existing datasets, such as NHANES, to provide a more comprehensive understanding of adolescent dietary patterns. By incorporating information on a wider range of foods, including those often excluded from traditional dietary assessments, citizen science data has the potential to enhance our understanding of the complex relationships between diet and health outcomes.

Moving forward, it will be important to continue exploring the link between nutritional patterns, demographic factors, and consumer behaviors in this population to better inform public health interventions aimed at improving dietary quality and reducing chronic disease risk.

## Data availability statement

The NHANES dataset is publicly available. The citizen science dataset in the study are not publicly available to protect the privacy of research participants, but aggregated datasets are available from the corresponding author on reasonable requests.

## Ethics statement

The studies involving humans were approved by the George Washington University Office of Human Research-Institutional Review Board. The studies were conducted in accordance with the local legislation and institutional requirements. Written informed consent for participation in this study was provided by the participants’ legal guardians/next of kin.

## Author contributions

QZ and JT: conceptualization and methodology. JT: formal analysis. JU, LZ, AL, and GT: resources. JU and JT: data curation. QZ, JT, and ST: writing—original draft preparation and writing—review and editing. QZ and JT: visualization. QZ and JU: supervision. QZ, LZ, TC, and GT: project administration. QZ, LZ, TC, AL, and GT: funding acquisition. All authors contributed to the article and approved the submitted version.
